# The Complexity of Antibody Responses Elicited against the Respiratory Syncytial Virus Glycoproteins in Hospitalized Children Younger than 2 Years

**DOI:** 10.3389/fmicb.2017.02301

**Published:** 2017-11-22

**Authors:** Alfonsina Trento, Rosa Rodríguez-Fernández, María I. González-Sánchez, Felipe González-Martínez, Vicente Mas, Mónica Vázquez, Concepción Palomo, José A. Melero

**Affiliations:** ^1^Unidad de Biología Viral, Centro Nacional de Microbiología, Madrid, Spain; ^2^CIBER de Enfermedades Respiratorias, Instituto de Salud Carlos III, Madrid, Spain; ^3^Hospital General Universitario Gregorio Marañón, Instituto de Investigación Sanitaria Gregorio Marañón – CIBEREHD, Madrid, Spain

**Keywords:** bronchiolitis, viral, respiratory syncytial virus infections, glycoproteins, antibody specificity, immune responses

## Abstract

The influence of age and maternal antibodies on the antibody responses to human respiratory syncytial virus (hRSV) glycoproteins in very young children has been a matter of controversy. Both, immaturity of the immune system at very early age and suppression of the host immune response by high level of maternal antibodies have been claimed to limit the host antibody response to virus infection and to jeopardize the use of hRSV vaccines under development in that age group. Hence, the antibody responses to the two major hRSV glycoproteins (F and G) were evaluated in children younger than 2 years, hospitalized with laboratory confirmed hRSV bronchiolitis. A strong negative correlation was found between the titre of circulating ELISA antibodies directed against either prefusion or postfusion F in the acute phase, but not age, and their fold change at convalescence. These changes correlated also with the level of circulating neutralizing antibodies in sera. As reported in adults, most neutralizing antibodies in a subset of tested sera could not be depleted with postfusion F, suggesting that they were mostly directed against prefusion-specific epitopes. In contrast, a weak negative association was found for group-specific anti-G antibodies in the acute phase and their fold change at convalescence only after correcting for the antigenic group of the infecting virus. In addition, large discrepancies were observed in some individuals between the antibody responses specific for F and G glycoproteins. These results illustrate the complexity of the anti-hRSV antibody responses in children experiencing a primary severe infection and the influence of preexisting maternal antibodies on the host response, factors that should influence hRSV serological studies as well as vaccine development.

## Introduction

Human respiratory syncytial virus (hRSV) is the main cause of severe acute lower respiratory tract infections (ALRI) in infants (bronchiolitis and pneumonia) and young children worldwide (Hall et al., 2009; Nair et al., 2010) Most severe hRSV infections occur in winter epidemics in temperate climates during the first year of life and more than 50% occur within the first 6 months (Glezen et al., 1986). Reinfections are common throughout life but they are ordinarily less severe (Hall et al., 2013). There are also apparent links between severe hRSV infection early in life and later development of asthma and wheeze (Blanken et al., 2013). hRSV is also an important cause of morbidity and mortality in the elderly and in adults with cardiopulmonary disease or with an impaired immune system (Falsey et al., 2005).

Human respiratory syncytial virus is an enveloped virus with a genome made of a negative single-stranded RNA that encodes 11 proteins, three of which are membrane-associated glycoproteins (G, F, and SH) (for a review, Collins and Melero, 2011). The two main glycoproteins of the hRSV virus particle are the G glycoprotein, initially dubbed as the receptor-binding or attachment protein (Levine et al., 1987) and the fusion (F) protein, identified by Walsh and Hruska (1983) as the protein responsible for fusion of the viral and cell membranes enabling entry of the virus ribonucleoprotein into the cell cytoplasm. SH is a small glycoprotein that is incorporated in low amounts into the virus particle and whose function remains largely unknown.

Human respiratory syncytial virus isolates were initially classified into two antigenic groups (A and B) based on reactivity with hyperimmune serum and later with G protein specific monoclonal antibodies (mAbs) (Anderson et al., 1985b; Mufson et al., 1985). Antigenic groups A and B were found to correlate with genetically distinct viral lineages (for a review, Melero and Moore, 2013). The G glycoprotein is a type II glycoprotein synthesized as a precursor of 297–310 amino acids (depending on the strain) that is heavily glycosylated with N-linked and O-linked oligosaccharides. The G protein ectodomain has a central conserved region, devoid of carbohydrates and that includes a cluster of four Cys residues, flanked by two highly variable mucin-like regions (Wertz et al., 1985). Conserved and group-specific epitopes cluster in the central conserved region of the G-protein ectodomain, whereas strain variable epitopes map preferentially within the C-terminal third of the G protein (Martinez et al., 1997; Melero et al., 1997).

The F glycoprotein is a type I fusion protein which is synthesized as a F0 precursor that requires proteolytic processing at two polybasic sites to become functional (Gonzalez-Reyes et al., 2001; Zimmer et al., 2001). hRSV F is a homotrimer that assembles in a metastable prefusion conformation in the virus particle before engaging in membrane fusion. During membrane fusion, the F glycoprotein refolds through a series of unstable intermediates into a highly stable postfusion conformation which shares some neutralizing epitopes with prefusion F (Lamb et al., 2006; McLellan et al., 2013b). hRSV F is highly conserved both at the antigenic and at the sequence level (Johnson and Collins, 1988), although some F-specific mAbs distinguish viruses of the two antigenic groups (McLellan et al., 2013c).

Neutralizing antibodies play a major role in protection against hRSV infections. For instance, passive transfer of immune serum protects mice (Graham et al., 1993) and cotton rats (Prince et al., 1985) against a hRSV challenge. In humans, high titres of serum neutralizing antibodies correlate with protection of adult volunteers against a hRSV challenge (Hall et al., 1991), and lower the risk of hRSV infection in children (Glezen et al., 1986) and in the elderly (Falsey and Walsh, 1998). It was recently found that most human neutralizing antibodies are specific of the prefusion conformation of hRSV F (Magro et al., 2012; Ngwuta et al., 2015), although antibodies that recognize epitopes shared by the prefusion and postfusion conformation of hRSV F can also neutralize virus infectivity (Magro et al., 2012) and protect cotton rats (Swanson et al., 2011) and mice (Palomo et al., 2016) against a virus challenge.

Despite the previous assertions, most severe hRSV infections occur very early in life when the level of trans-placentally transferred maternal antibodies is still high (Chu et al., 2014). This has raised the question whether certain unidentified antibody characteristics (e.g., epitope specificity or neutralization potency) may be responsible for the failure to protect some children against severe hRSV infection (Jans et al., 2017). In addition, it has been suggested that the presence of maternal antibodies may have an immunosuppressive effect on the infant’s own immune response and that immaturity of the immune system may also contribute to a poor response in very young children (Murphy et al., 1986). However, Shinoff et al. (2008) reported that a significant fraction of Navajo and White Mountain Apache children aged 0–24 months hospitalized with hRSV infections developed a neutralization antibody response. Multivariable analysis indicated that the level of pre-existing antibodies, not age, was the most important factor influencing the neutralizing response. Therefore, careful characterization of antibodies and antibody responses in very young children may inform on the protective efficacy (or failure) of certain antibodies and the capacity of the infant immune system to respond to hRSV infections. Hence, we evaluated ELISA binding antibodies to the F and G glycoproteins and neutralizing antibody responses in hospitalized children with laboratory confirmed bronchiolitis. Highly significant negative correlations were found between the level of pre-existing ELISA binding antibodies -particularly to the prefusion and postfusion forms of hRSV F- and the extent of the antibody response. As in adults, most of the infant neutralizing antibodies could not be depleted with postfusion F, suggesting that they recognized prefusion specific epitopes. Unexpectedly, highly discrepant antibody responses to the F and G glycoproteins were found in some children, adding unanticipated complexity to the infant’s antibody response.

## Materials and Methods

### Study Design and Clinical Samples

Children less than 2 years old were hospitalized in the infant ward of “Hospital General Universitario Gregorio Marañón” (HGUGM, Madrid, Spain) during the 2007–2008, 2012–2013, and 2013–2014 epidemics with hRSV infections confirmed using the Alere BinaxNow^®^ RSV quick test on nasopharyngeal samples. Patients were excluded from study if they had a gestational age <35 weeks; congenital heart disease, chronic lung disease, or history of immunodeficiency. None of the patients received prophylaxis with palivizumab. Severity of RSV bronchiolitis was assessed with different parameters including length of stay (LOS), days of oxygen, and modified wood downes severity score. Severity score was assessed at the time of admission and ranged from 1 to 14 (mild bronchiolitis:1–3, moderate:4–7, severe:8–14). Nasopharyngeal aspirates and serum samples were taken from patients at admission (acute phase). Serum samples were also taken one month later (convalescent phase). Aspirates and serum samples were stored in the hospital BioBank at -80°C until transfer to laboratory premises for analysis. Informed consent was obtained from the patients’ parents or guardians. The experimental protocols used in the study were review and endorsed by the “Cómite de Ética de la Investigación y del Bienestar Animal” CEI PI 48_2015-v3, del Instituto de Salud Carlos III.

### Sequencing and Phylogeny of the G Protein Gene

RNA extraction from the nasopharyngeal aspirates as well as amplification and sequencing of the G protein gene were performed as described (Trento et al., 2015). Phylogenetic trees were built with the maximum-likelihood method, using the MEGA software version 6.0 (Tamura et al., 2013). Sequences representative of the major clades within hRSV A and hRSV B antigenic groups were included in the phylogenetic analysis. The new sequences reported here that correspond to group B viruses from Madrid were deposited in GenBank database with accession numbers MF443140 to MF443158. The remaining sequences used in this study were downloaded from GenBank and their accession numbers are listed in Supplementary Table S2.

### Virus Isolation

Nasopharyngeal aspirates were also used to infect HEp-2 cell monolayers growing in Dulbecco’s modified Eagle’s medium supplemented with 2% fetal calf serum (DMEM2). Cytopathic effect was monitored by light microscopy and when it was visible (usually 3–4 days after infection), cells were scrapped into the medium and thoroughly suspended in culture supernatants which were stored in liquid N2 until use. After thawing, virus stocks were spun down at low speed to remove cell debris before being used to infect new cell cultures. Manipulations of samples, cells and viruses were confined to biosafety level 2 laboratories.

### Fusion Proteins and F Protein Specific ELISAs

Production, purification and characterization of soluble forms of the hRSV F protein (Long strain), either stabilized in the prefusion conformation (Pre-F), or refolded in the highly stable postfusion form (Post-F), have been described (Palomo et al., 2016). Stabilization of the Pre-F conformation was based on the strategy used by McLellan et al. (2013a) for the generation of the DS-Cav1 protein. Both Pre-F and Post-F have the fold-on trimerization domain added at the C-terminus of the F protein ectodomain (Meier et al., 2004), followed by a 6-His tag to facilitate purification in Ni2+ columns, as described (Mas et al., 2016).

The following ELISA format was used to test reactivity of soluble F proteins with serum antibodies: Two hundred nanograms of a proprietary anti-foldon mAb (MF4) in 50 μl of PBS were used to coat 96-well plates overnight at 4°C. After blocking with 2% pig serum in phosphate buffer saline (PBS) containing 0.05% Tween 20 (150 μl), 40 ng of purified Pre-F or Post-F were added and incubation continued for one hour at 37°C. After washing with water, serum dilutions were added and bound antibodies revealed with horseradish peroxidase-labeled anti-human Ig (50 μl) and *O-*phenyl-diamine (OPD) as substrate, following manufacturer’s instructions (GE Healthcare). Reactions were stopped with 2N H_4_SO_2_ (50 μl) and color read at 492 nm.

### G Protein Specific ELISAs

HEp-2 cell monolayers were infected with 1–3 plaque forming units/cell (pfu/cell) of the viruses MAD/GM2_14/13 from antigenic group A or BA/3833/99 from antigenic group B using Dulbecco’s medium supplemented with 2% fetal calf serum and antibiotics (DMEM2), as described (Garcia-Barreno et al., 1989). Forty-eight hours later, cells were scrapped off, washed by low speed centrifugation, and solubilized in extraction buffer (10 mM Tri-HCl, pH 7.6, 5 mM EDTA, 140 mM NaCl, 1% octyl-glucoside). Extracts were clarified in a minifuge at maximum speed for 5 m before being used.

For ELISAs, two hundred nanograms of the mAb 021/1G, which recognizes a fully conserved G protein epitope (Martinez et al., 1997), were used to coat 96-well plates overnight at 4°C. After blocking, as described in the previous section, a predetermined amount of infected cell extracts diluted in blocking buffer (50 μl) were added to the wells and the incubation continued for one hour at 37°C. After washing with water, serum dilutions were added and bound antibodies revealed as described above.

#### Virus Neutralization

Two different assays were used. The first assay was based on the microneutralization test of Anderson et al. (1985a) as previously described (Magro et al., 2010). Briefly, dilutions of human sera were incubated with 2 × 10^2^ pfu of virus for 30 min at room temperature in a total volume of 50 μl. These mixtures were used to infect 5 × 10^4^ HEp-2 cells growing in 96-well plates with DMEM2. After 1 h of adsorption, 150 μl of DMEM2 were added and incubation continued for 72 h at 37°C. Then, the plates were washed three times with 0.05% Tween 20 in PBS and fixed with 80% cold acetone in PBS. After air drying, viral antigen production in the fixed monolayers was measured by ELISA with a pool of anti-G (021/1G and 021/21G) and anti-F (47F, 101F, 56F, and 2F) murine MAbs (Palomo et al., 2016), essentially as described in the previous section.

The second neutralization assay made use of a recombinant hRSV (A2 strain) that expresses the green fluorescent protein [hRSV/GFP (Hallak et al., 2000), kindly provided by Mark Peeples (Nationwide Children’s Hospital, Columbus, OH, United States]. Serum dilutions were incubated for 30 min at 37°C with a predetermined amount of hRSV/GFP before being added to monolayers of HEp-2 cells growing in 96-well plates in a total volume of 150 μl of DMEM2. Forty-eight hours later, the medium was removed and after washing twice with PBS, fluorescence was measured in a Tecan microtitre reader M200.

#### Depletion of Postfusion F Specific Antibodies

It was done essentially as described by Magro et al. (2012). Briefly, antibodies from individual sera were purified with protein A-Sepharose columns, as recommended by the manufacturer (GE Healthcare). The purified antibodies were loaded onto a Sepharose column with covalently linked purified hRSV postfusion F protein (Long strain). The unbound material containing the postfusion F depleted antibodies was collected and saved for later use.

### Statistical Analysis

Statistical analysis was performed using Prism Graphpad v6 and SPSS 21.0 statistical package (SPSS Inc., Chicago, IL, United States). Quantitative variables were expressed as means and standard deviations (mean ± SD) or medians and interquartile range (IQR) and qualitative variables were expressed as percentages. Chi square test and Fisher exact test were used to compare qualitative variables and Mann–Whitney *U* test and Kruskall Wallis test were used to compare two or more groups of quantitative variables. The relationship between quantitative variables was examined using Pearson or Spearman’s correlation.

Linear regression models were built using antibody fold change as dependent variable, and the covariates introduced in the models were age and acute antibody levels. Goodness of fit of our final set of predictors to fold change was expressed as the adjusted *R*^2^. The assumption of no multicollinearity was evaluated in all models. A *p*-value < 0.05 was considered statistically significant.

## Results

### Virus Samples

Nasopharyngeal aspirates were obtained from 44 children at the time of hospitalization during the winters of 2007–2008, 2012–2013, and 2013–2014. Full-length G protein gene sequences could be amplified directly from all clinical samples, hence confirming the results of the Alere BinaxNow^®^ RSV quick test. Although no quantitative estimation was made, the presence of hRSV sequences in the respiratory tract was indicative of substantial viral loads at the time of hospitalization. The G sequences were used to build phylogenetic trees in which representatives of the main group A and group B genotypes were also included (Supplementary Figure S1). As noted in many other studies, dominance of group A and group B virus alternated in different seasons. Thus, while a slight dominance of group A (9 out of 15 samples) over group B viruses was observed in 2007–2008, only group A viruses were detected in 2012–2013 and group B viruses were highly dominant in 2013–2014 (13 out of 15 samples). Sequences of the clinical samples clustered in the phylogenetic trees with those from prototypic genotypes circulating at the time of sampling. For instance, group A viruses from 2007 to 2008 clustered into genotypes GA5 and GA2 and lacked the 72-nucleotide duplication (ON1) first detected in Ontario in 2010–2011 (Eshaghi et al., 2012) whereas group A viruses from 2012 to 2013 and 2013 to 2014 exhibited this duplication that has become dominant in recent years (Agoti et al., 2014). Similarly, group B viruses had the 60-nucleotide duplication (Trento et al., 2003) characteristic of the BA genotype, which has replaced globally other group B genotypes since mid-2000’s (Trento et al., 2010). In brief, none of the viruses analyzed in this study showed characteristics dissimilar from those of circulating viruses that could affect the conclusions about antibody responses reached in succeeding sections.

### Glycoprotein Binding and Neutralizing Antibodies

**FIGURE 1:**
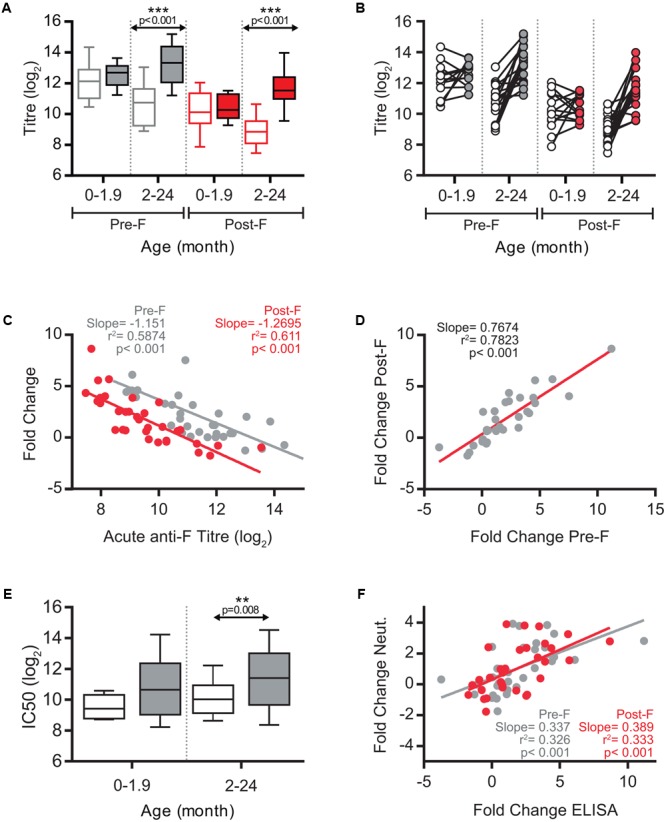
ELISA antibody response against hRSV F and neutralization response. **(A)** Box-and-whisker plots showing ELISA antibodies titres against Pre-F (gray) and Post-F (red) proteins, in acute (empty boxes), and convalescent (filled boxes) phases of infection. The boxes represent the first and the third quartiles, and the solid horizontal lines within the box represent the mean values. The whiskers represent lowest and highest values. **(B)** Relationship between individual acute and convalescent ELISA titres against Pre-F and Post-F glycoproteins. **(C)** Scatterplots comparing acute anti-Pre-F and anti-Post-F antibodies titres vs fold change of antibody titres between acute and convalescent phase. Correlation coefficient *R*^2^, slope, and *p*-value are shown. **(D)** Scatterplot of individual sera comparing titre fold change for Pre-F protein vs. titre fold change for Post-F protein. **(E)** Box-and-whisker plots showing hRSV neutralizing antibody titres in acute (empty box) and convalescent (gray box) phases of infection. Neutralization was done as described in Section “Materials and Methods” using the recombinant A2 strain of hRSV that expresses GFP. **(F)** Scatterplots of individual sera comparing fold changes of ELISA titres for Pre-F and Post-F proteins versus fold change in neutralization titre.

A total of 33 paired serum samples were available from children with ages ranging from 10 days to 17 months at the time of hospitalization (Supplementary Table S1). Clinical and pathological parameters were also recorded, as shown in Supplementary Table S1. There were almost equal numbers of females (17) and males (14). Severity scores fluctuated between 4 and 9 in a scale of 1–14. There was only one co-infection detected with cytomegalovirus without specific symptoms or analysis results to set it apart from other samples. Acute serum samples were taken at admission and convalescent samples one month later. Acute and convalescent sera were firstly tested for ELISA binding antibodies, using as antigens purified soluble forms of the hRSV F glycoprotein (Long strain) stabilized in either its prefusion (Pre-F) or postfusion (Post-F) conformation (Rodriguez et al., 2015). Individual ELISA titres are shown in Supplementary Table S1 and summarized in **Figure 1**. Sera were split into two age groups (<2 and >2 two months) that distinguish best the antibody responses. The mean titre of acute phase antibodies binding to prefusion F was higher in the <2 months group than in the >2 months group (**Figure 1A**). This is likely due to higher titres of maternal antibodies at younger age. In addition, the increase of mean antibody titre in convalescent versus acute sera was not significant in the younger group whereas it was highly significant in the older group (*p* < 0.001) (**Figure 1A**). The same trend was observed with antibodies that bound to postfusion F (**Figure 1A**). However, the mean titre analysis of **Figure 1A** blurred sizeable changes discernable when acute and convalescent sera from the same individual were directly compared (**Figure 1B**). Thus, in children <2 months some ELISA titres increased at convalescence while others decreased, despite no significant differences at the group level (**Figure 1A**). Similarly, large changes in ELISA titres were observed in individuals >2 months whereas in other individuals the titre remained essentially unchanged at convalescence.

Motivated by the report of Shinoff et al. (2008) a regression analysis was performed for the ELISA antibody titre in the acute phase and the increase in antibody titre after convalescence. The analysis shown in **Figure 1C** demonstrated a highly significant negative correlation between the antibody levels against either prefusion or postfusion F in the acute phase and the fold change in antibody titre one month later. In a multivariate analysis in which the acute level of anti-F antibodies and age were included as covariates (**Table 1**), only the anti-F antibody titre in the acute phase, and not age, correlated with the fold change of antibody titre at convalescence, suggesting that the basal level of antibodies dominated the extent of the anti-F antibody response. This response was similar for antibodies that bound to either prefusion or postfusion F as shown by the correlation of their respective fold changes (**Figure 1D**); i.e., no individual response targeted predominantly prefusion or postfusion specific epitopes.

**Table 1:** Multivariable correlations between fold change of antibodies titer between acute and convalescent phases and relevant variables.

Dependent	Independent	Regression	Std.	*P*	*R*^b^	Adjusted
variable^a^	variable	coefficient (95%CI)	Error			*R*^2b^
*Fold change Pre-F*					*0.826*	*0.659*
	Acute anti-Pre-F	–1.150 (-1.558 to-0.741)	0.199	***<0.001***		
	Age (month)	–0.05 (-0.156 to 0.145)	0.073	0.941		
*Fold change Post-F*					*0.833*	*0.671*
	Acute anti-Post-F	–1.236 (-1.615to-0.858)	0.185	***<0.001***		
	Age (month)	0,101 (0.022 to 0.224)	0.060	0.104		
*RSV A infected*					*0.496*	*0.413*
Fold change anti-GA	Acute anti-GA	–0.816(-1.540 to-0.093)	0.332	***0.030***		
	Age (month)	–0.003 (-0.399 to 0.394)	0.182	0.989		
*RSV A infected*					*0.418*	*0.320*
Fold change anti-GB	Acute anti-GB	–0.282 (-0.884 to 0.320)	0.276	0.327		
	Age (month)	–0.196 (-0.069 to 0.460)	0.121	0.133		
*RSV B infected*					*0.320*	*0.261*
Fold change anti-GA	Acute anti-GA	–0.472 (-0.835 to-0.108)	0.171	***0.014***		
	Age (month)	–0.060 (-0.218 to 0.097)	0.074	0.429		
*RSV B infected*					*0.521*	*0.457*
Fold change anti-GB	Acute anti-GB	–0.751 (-1.259 to-0.244)	0.238	***0.007***		
	Age (month)	0.054 (-0.175 to 0.284)	0.108	0.621		

***^a^**Dependent variables: fold changes in the titres of the indicated antibodies. **^b^**Multiple regression analysis summary. All covariates were analyzed as continuous variables. Bold type indicates parameters that were significantly different in adjusted analysis. CI: confidence interval.*

A similar trend to that of anti-F binding antibodies (**Figure 1A**) was found for neutralizing antibodies (**Figure 1E**), although the magnitude of the fold change between acute and convalescent sera was lower for neutralizing than for binding antibodies. A highly significant correlation was observed between the fold change in neutralizing and ELISA binding antibodies (**Figure 1F**), indicating that the individual antibody responses were not skewed for either neutralizing or non-neutralizing antibodies.

**FIGURE 2:**
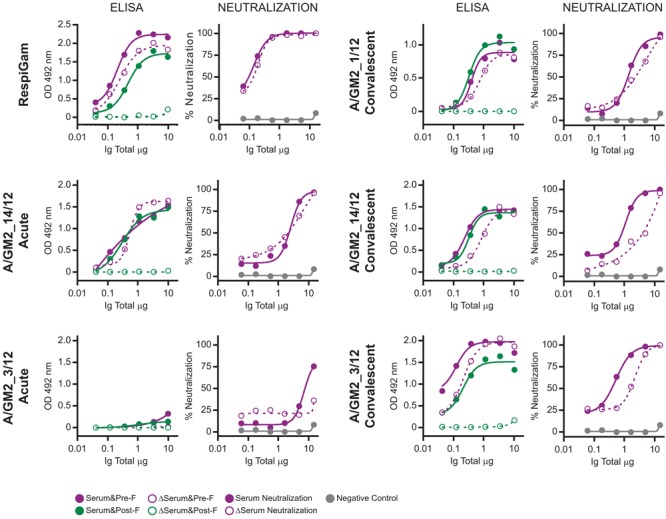
ELISA and neutralization before and after depletion of antibodies binding to postfusion hRSV F. Antibodies from the indicated serum samples were purified with protein A-Sepharose and processed in parallel with RespiGam for depletion of antibodies binding to postfusion hRSV F, as described (Magro et al., 2012). For each serum sample, the left panel shows the ELISA titration of antibodies binding to Pre-F (red) and Post-F (green) proteins before (fill circles) and after (empty circles) the depletion step and the right panel the neutralization of hRSV either before (fill circles) or after depletion (empty circles) of antibodies binding to postfusion F.

It has been reported that in adults the majority of hRSV neutralizing antibodies recognize epitopes specific of the F glycoprotein folded in its prefusion conformation (Magro et al., 2012; Ngwuta et al., 2015). To test if this was also true in children, a subset of sera -from which enough volume was available- were processed for depletion of antibodies binding to postfusion F, as previously described (Magro et al., 2012). As reference control, RespiGam (a commercial human Ig preparation made from donors with high titres of neutralizing anti-hRSV antibodies, Groothuis et al., 1993) was processed in parallel. As published before, RespiGam antibodies depleted of those binding to postfusion F (confirmed by ELISA, see **Figure 2**) retained most of their capacity to bind to prefusion F (see ELISA panel) and neutralized hRSV almost as efficiently as before depletion (see neutralization panel). Serum A/GM2_1/12, from which only the convalescent sample was available behaved similarly (**Figure 2**); i.e., antibodies depleted of those binding to postfusion F still bound to prefusion F (ELISA) and neutralized the virus (neutralization) almost as efficiently as the initial sample. The A/GM2_14/12 antibodies, obtained from either acute or convalescent sera, behaved similarly to convalescent A/GM2_1/12. Interestingly, A/GM2_3/12 (**Figure 2**, lower panels) showed a sharp increase in anti-F antibody titres after convalescence (**Figure 2**, lower panels), indicative of a strong host antibody response to the virus although this child was only 7 months old. Nonetheless, depletion of antibodies binding to postfusion F in convalescent serum had minimal impact on neutralization (**Figure 2**, lower right panel), mimicking the results obtained with adult Ig, such as RespiGam.

**FIGURE 3:**
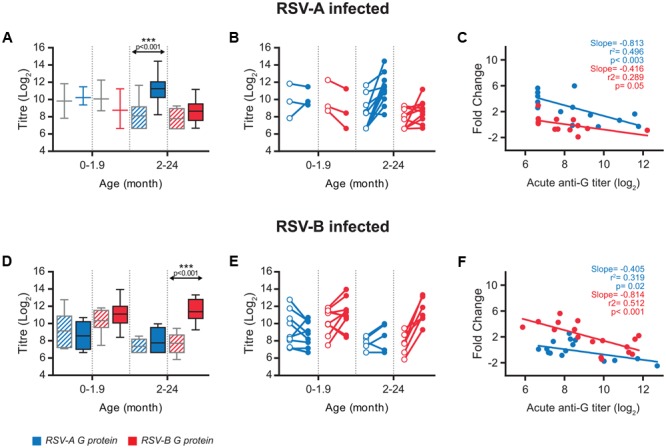
Antibody response against G protein. **(A,D)** Box-and-whisker plots showing antibody ELISA titres against homologous and heterologous G protein, in acute (grated pattern box) and convalescent (filled box) phase of infection. The boxes represent the first and the third quartile, and the solid horizontal lines within the box represent the mean values. The whiskers represent the lowest and highest values. Antibody titters against RSV-A G protein are colored in blue and antibody titres against RSV-B G protein are colored in red. **(B,E)** Relationship between individual acute and convalescent titres against the G glycoprotein. **(C,F)** Scatterplots comparing acute anti-G antibodies titres vs. fold change at convalescence. Correlation coefficient *R*^2^, slope and *p*-value are shown.

The antibodies directed against the other major hRSV glycoprotein (G) were also quantified in the acute and convalescent serum samples (Supplementary Table S1). Since the level of sequence divergence between the G proteins of antigenic group A and B viruses is very high (∼50%) (Johnson et al., 1987), sera were tested for antibodies binding to G proteins representative of group A and B viruses (GA and GB). Interestingly, always that an increase in the level of anti-G antibodies was observed at convalescence it was higher for the protein from the same antigenic group as the virus present in the nasopharyngeal aspirate than for the heterologous protein (Supplementary Table S1). These results are summarized in **Figure 3**. No significant increase of mean antibody titres after convalescence was observed in children younger than two months, although the number of children infected with group A virus was too small (*n* = 3) to reach definitive conclusions. In contrast, a significant but group specific response was noted in children 2-24 months of age. Thus, mean ELISA titres increased significantly against GA, but not GB, in children infected with group A viruses (**Figure 3A**) and reciprocally a significant increase in antibodies directed against GB, but not GA, was noted in children infected with group B viruses (**Figure 3D**). As for anti-F antibodies, sizeable changes could be observed at the individual level when acute versus convalescent anti-G serum titres were compared (**Figures 3B,E**). Again, most anti-G antibody responses were observed in children 2–24 months of age and were almost always specific for the antigenic group of the infecting virus (Supplementary Table S1).

In contrast with anti-F antibodies, relatively weak negative correlations (lower slope values) were found between anti-G antibody levels in the acute phase and fold change at convalescent (**Figures 3C,F**, compare with **Figure 1C**). This correlation was stronger (steeper slope) and more statistically significant (lower p value) when titres against the G protein of the same antigenic group as the infecting virus were considered. In a multivariate analysis, including basal anti-G antibodies and age (**Table 1**), group-specific acute anti-GA and anti-GB antibody titres correlated significantly with fold change at convalescence, but not with age. This correlation was also statistically significant for acute anti-GA antibodies in children infected with group B viruses and fold change of antibody titres at convalescence.

**FIGURE 4:**
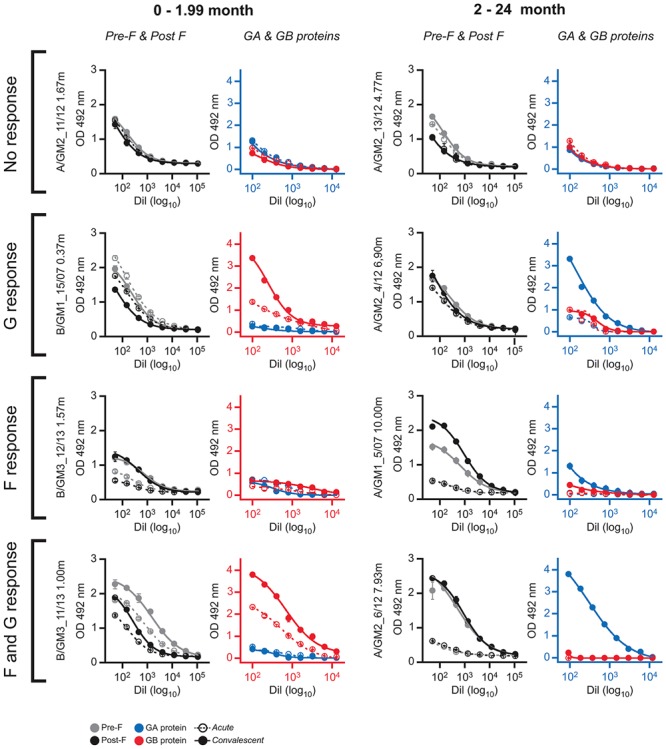
ELISA antibody responses against F and G glycoproteins in selected individual serum samples. The serum samples are indicated at left with the nomenclature of Supplementary Table S1. The left panels of each serum sample show the ELISA antibody titrations against Pre-F and Post-F fusion proteins. The right panels are the ELISA titrations of antibodies against G_A_ and G_B_ glycoproteins. Axes in each panel are colored red or blue according to the antigenic group (blue, group A and red, group B) of the virus that infected each individual.

A remarkable feature of the results shown in Supplementary Table S1 is the lack of correlation in some children between the antibody responses elicited against F and G glycoproteins. Representative examples are shown in **Figure 4**. There were cases in which antibody levels against both F and G remained unchanged at convalescence (**Figure 4**, no response). In contrast, some individuals responded against G but not against F (**Figure 4**, G response) or vice versa (**Figure 4**, F response). Finally, in some cases significant antibody titres against both F and G were observed at the convalescent phase (**Figure 4**, F and G response). The discrepant situations, however, were apparently not influenced by age or the antigenic group of the infecting virus.

**FIGURE 5:**
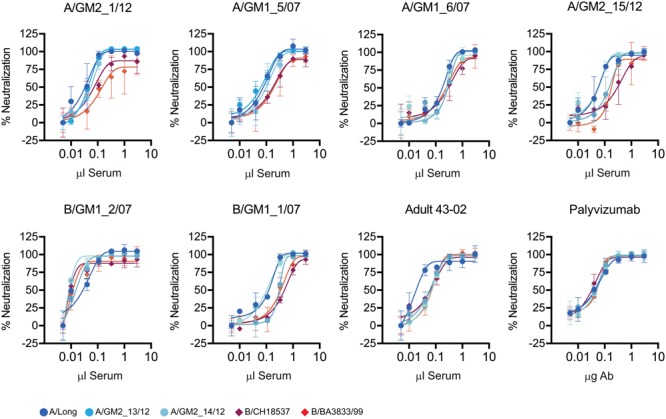
Virus neutralization. Each panel shows the neutralization curves of a set of hRSV group A and B virus by the sera indicated at the top of each panel. Neutralization was performed by the neutralization test described in Section “Materials and Methods”. As controls, serum from an adult volunteer (43-02) and the monoclonal antibody Palivixumab are shown in the two lower right panels.

To assess if the noted group specificity of anti-G antibody responses in children had an impact on neutralization, a selected subset of six convalescent sera were tested for neutralization of three viral strains of antigenic group A and two strains of antigenic group B, including some of the viruses isolated from the clinical samples (A/GM2_13/12, A/GM2_14/12) and historical viruses (A/Long, B/CH18537 and B/BA3833/99) (**Figure 5**). All sera neutralized the five viral strains irrespective of the antigenic group of the infecting virus; i.e., neutralization mediated by children sera did not correlate with the group antibody response of ELISA antibodies binding to the G glycoprotein.

Finally, we analyzed if there was any association between clinical parameters shown in Supplementary Table S1 and antibody concentrations. We did not find any correlation between acute or convalescent antibody concentration and length of stay (LOS) in hospital, severity score or days of oxygen (*p* > 0.05). Neither have we found any differences in basal antibody concentrations against F and G proteins depending on whether the patient was being breastfed or not.

## Discussion

It has been generally found in different reports a paucity of the specific antibody response in very young children (0–6 months old) compared with older children infected with hRSV (Murphy et al., 1986; Sande et al., 2014). This age-related effect was attributed to either immaturity of the immune system or the higher level of maternal suppressive antibodies in very young infants or both (for a review, Crowe, 2001). In agreement with the results reported by Shinoff et al. (2008) for neutralizing antibodies, we found that the magnitude of the antibody response against the F glycoprotein (both prefusion and postfusion forms) was negatively associated with the antibody level in the acute phase and not with age. This immunosuppressive effect of preexisting (presumably maternal) antibodies mimics the reported suppression of the primary humoral response against attenuated hRSV vaccines in mice passively inoculated with anti-hRSV antibodies (Crowe et al., 2001). The higher level of maternal anti-F antibodies at younger age (**Figure 1A**) may have had the confounded effect ascribed in other studies to age.

Although changes in ELISA anti-F antibodies correlated with changes in neutralizing antibodies (**Figure 1F**), the former were notably of higher magnitude. Hence, F specific ELISA tests may be more sensitive than neutralization assays to evaluate the antibody response and for serologic diagnostics of hRSV infections.

The antibody responses against the G glycoprotein were generally weaker than against F and were mostly group-specific. This group-specificity (Hendry et al., 1988) is likely due to antibodies elicited against the central unglycosylated region of the G glycoprotein, as noted in adults infected with hRSV (Murata et al., 2010a,b) or in mice challenged i.n. with the virus (Murata and Catherman, 2012). However, the group-specific response noted in ELISA with the G glycoprotein was not reflected in group-specific neutralization of viruses, in agreement with the dominance of cross-reactive anti-F antibodies -particularly those specific for prefusion F epitopes- in the human neutralizing response (Magro et al., 2012; Ngwuta et al., 2015).

In the few samples in which depletion studies could be performed, the majority of the neutralizing activity was not eliminated, as in adults, by removing the antibodies binding to postfusion F, even in the case of a significant host response without pre-existing maternal antibodies in the acute phase (sample A/GM2_3/12, **Figure 2**). Therefore, the dominance of neutralizing prefusion specific epitopes is a trait of the human antibody response from a very early age.

Hendry et al. (1988) and Sande et al. (2013) reported certain group-specificity of the neutralizing antibody response in infants and young children after primary hRSV infection, although extensive cross-neutralization of viruses of the heterologous antigenic group was also apparent in their studies. Given the dominance of cross-reactive anti-F in the neutralizing response, the apparent contribution of anti-G antibodies that might confer certain group-specific neutralization requires further investigation. It is worth mentioning that broader cross-neutralization has been noted in sera of children after secondary hRSV infection with heterologous virus than after primary infection (Muelenaer et al., 1991). In any case, as in previous studies (Sande et al., 2013), we observed neutralization irrespective of whether the test virus was contemporary or historical, questioning the role played by neutralizing antibodies on hRSV evolution (Trento et al., 2015).

One of our intriguing findings was the lack of correlation in certain children between the antibody responses to the F and G glycoproteins (**Figure 4**). This discrepancy may have been overlooked in other studies that grouped together antibody titres without individual analysis. The reason for this discrepant response to glycoproteins that are present in the same virus particle is unclear and deserves further investigation. It is worth mentioning in this context the reported differences in subclass immunoglobulins to F and G glycoproteins (Wagner et al., 1986, 1987), owing to the high carbohydrate content of G which resembles a polysaccharide antigen. It is thus possible that certain genetic backgrounds may respond differently to protein-like versus carbohydrate-like antigens.

As mentioned in the Introduction, paradoxically the incidence of severe hRSV infections is higher in the age group (0–6 months) with the highest level of maternal neutralizing antibodies. This is contradictory with prevention of severe hRSV infections by passive prophylactic administration to high risk children of neutralizing Igs (RespiGam) (Groothuis et al., 1993) or mAbs (Palivizumab) (Groothuis and Nishida, 2002). Certain studies have, however, failed to find an association between level of maternal antibodies and protection against severe hRSV infection (Nyiro et al., 2016). In this sense, it will be important to address in future studies some of the limitations of our current analysis; for instance, evaluation not only of antibody levels but assessment of antibody isotypes and epitope specificities in larger population samples may unveil unanticipated deficiencies associated with disease severity. Similarly, examination of viral load or innate and cellular immune responses may disclose parameters that correlate better with either disease severity or immune mediated protection. At any rate, our present study describes assays, reagents and initial data that should facilitate future investigations on immune mediated protection against hRSV infection.

## Author Contributions

AT, RR-F, and JM designed the study. RR-F, MG-S, and FG-M were responsible of all clinical aspects, including isolation of serum and nasopharyngeal aspirates and evaluation of disease progression, symptoms, and severity. AT isolated viruses from respiratory specimens and performed sequence and phylogenetic studies. AT, MV, and VM evaluated ELISA binding and neutralizing antibodies. MV and CP performed antibody depletion studies. AT, RR-F, and JM analyzed results, including statistical analysis. JM supervised the project and together with AT and RR-F wrote the manuscript. All authors discussed the results and commented on the manuscript.

## Conflict of Interest Statement

The authors declare that the research was conducted in the absence of any commercial or financial relationships that could be construed as a potential conflict of interest.
