# Supplementary material for: The Complexity of Antibody Responses Elicited against the Respiratory Syncytial Virus Glycoproteins in Hospitalized Children Younger than 2 Years
Source: Front Microbiol. 2017 Nov 22;8:2301. doi: 10.3389/fmicb.2017.02301 (PMC5702767; doi:10.3389/fmicb.2017.02301)
Supplement: Supplementary file 2 [file Table_2.DOCX]

| **Table S2. Accession numbers of sequences downloaded from GenBank used in this study** | |
| --- | --- |
| ACCESION NUMBERS | REFERENCE |
| **RSV-A** |  |
| M17212 | ([Johnson et al., 1987a](#_ENREF_2)) |
| KF300971, KF300981, KF300994, KF301013, KP792352-KP792376 | ([Trento et al., 2015](#_ENREF_6)) |
| Z33411, Z33416, Z33414, Z33420, Z33426, Z33428, Z33494, Z33431 | ([Garcia et al., 1994](#_ENREF_1)) |
| KF826840, KF826838 | § |
| JX069800 | § |
| **RSV-B** |  |
| M17213 | ([Johnson et al., 1987b](#_ENREF_3)) |
| AF013254 | ([Karron et al., 1997](#_ENREF_4)) |
| AB175819, AB175820 | ([Sato et al., 2005](#_ENREF_5)) |
| AY333362, AY333363 | ([Trento et al., 2003](#_ENREF_8)) |
| AY751105, AY751117 | ([Zlateva et al., 2005](#_ENREF_10)) |
| DQ227376, DQ227377, DQ227387, DQ227393 | ([Trento et al., 2006](#_ENREF_9)) |
| GQ150696, GQ150714, GQ150729, GQ150734, GQ150738, GQ150741 | ([Trento et al., 2010](#_ENREF_7)) |

§Direct submission to GenBank database. Unpublished data

Reference List

Garcia, O., Martin, M., Dopazo, J., Arbiza, J., Frabasile, S., Russi, J., et al. (1994). Evolutionary pattern of human respiratory syncytial virus (subgroup A): cocirculating lineages and correlation of genetic and antigenic changes in the G glycoprotein. *J Virol* 68(9)**,** 5448-5459.

Johnson, P.R., Spriggs, M.K., Olmsted, R.A., and Collins, P.L. (1987a). The G glycoprotein of human respiratory syncytial viruses of subgroups A and B: extensive sequence divergence between antigenically related proteins. *Proc.Natl Acad.Sci U.S.A* 84(16)**,** 5625-5629.

Johnson, P.R., Spriggs, M.K., Olmsted, R.A., and Collins, P.L. (1987b). The G glycoprotein of human respiratory syncytial viruses of subgroups A and B: extensive sequence divergence between antigenically related proteins. *Proc Natl Acad Sci U S A* 84(16)**,** 5625-5629.

Karron, R.A., Buonagurio, D.A., Georgiu, A.F., Whitehead, S.S., Adamus, J.E., Clements-Mann, M.L., et al. (1997). Respiratory syncytial virus (RSV) SH and G proteins are not essential for viral replication in vitro: clinical evaluation and molecular characterization of a cold-passaged, attenuated RSV subgroup B mutant. *Proc Natl Acad Sci U S A* 94(25)**,** 13961-13966.

Sato, M., Saito, R., Sakai, T., Sano, Y., Nishikawa, M., Sasaki, A., et al. (2005). Molecular epidemiology of respiratory syncytial virus infections among children with acute respiratory symptoms in a community over three seasons. *J Clin Microbiol* 43(1)**,** 36-40. doi: 10.1128/JCM.43.1.36-40.2005.

Trento, A., Abrego, L., Rodriguez-Fernandez, R., Gonzalez-Sanchez, M.I., Gonzalez-Martinez, F., Delfraro, A., et al. (2015). Conservation of G-Protein Epitopes in Respiratory Syncytial Virus (Group A) Despite Broad Genetic Diversity: Is Antibody Selection Involved in Virus Evolution? *J.Virol.* 89(15)**,** 7776-7785.

Trento, A., Casas, I., Calderon, A., Garcia-Garcia, M.L., Calvo, C., Perez-Brena, P., et al. (2010). Ten years of global evolution of the human respiratory syncytial virus BA genotype with a 60-nucleotide duplication in the G protein gene. *J Virol* 84(15)**,** 7500-7512. doi: 10.1128/JVI.00345-10.

Trento, A., Galiano, M., Videla, C., Carballal, G., Garcia-Barreno, B., Melero, J.A., et al. (2003). Major changes in the G protein of human respiratory syncytial virus isolates introduced by a duplication of 60 nucleotides. *J Gen Virol* 84(Pt 11)**,** 3115-3120. doi: 10.1099/vir.0.19357-0.

Trento, A., Viegas, M., Galiano, M., Videla, C., Carballal, G., Mistchenko, A.S., et al. (2006). Natural history of human respiratory syncytial virus inferred from phylogenetic analysis of the attachment (G) glycoprotein with a 60-nucleotide duplication. *J Virol* 80(2)**,** 975-984. doi: 10.1128/JVI.80.2.975-984.2006.

Zlateva, K.T., Lemey, P., Moes, E., Vandamme, A.M., and Van Ranst, M. (2005). Genetic variability and molecular evolution of the human respiratory syncytial virus subgroup B attachment G protein. *J Virol* 79(14)**,** 9157-9167. doi: 10.1128/JVI.79.14.9157-9167.2005.
