# Supplementary figures and images for: The Complexity of Antibody Responses Elicited against the Respiratory Syncytial Virus Glycoproteins in Hospitalized Children Younger than 2 Years
Source: Front Microbiol. 2017 Nov 22;8:2301. doi: 10.3389/fmicb.2017.02301 (PMC5702767; doi:10.3389/fmicb.2017.02301)

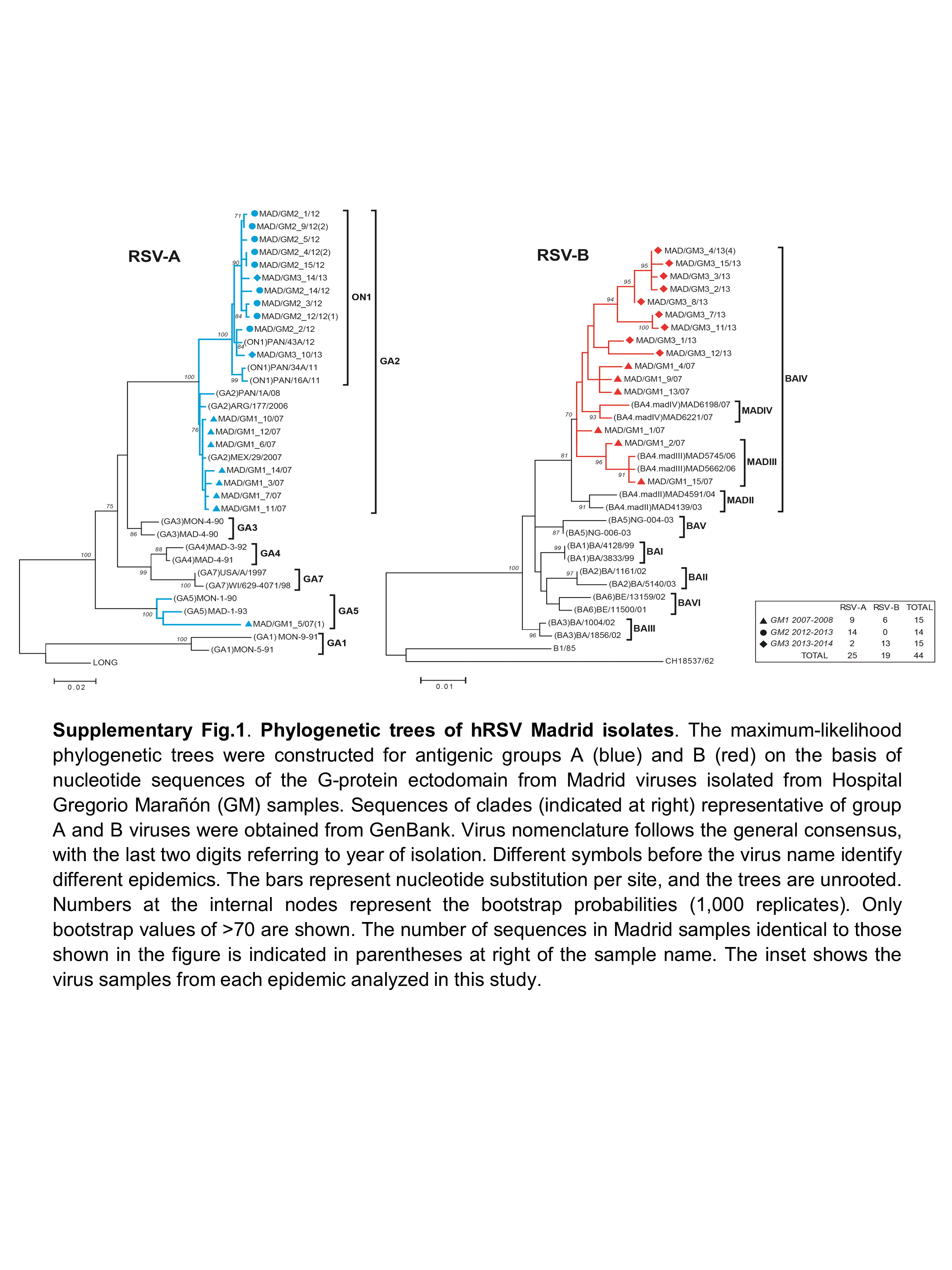

Supplement: Supplementary file 3 [file Image_1.TIF]
